# In Silico Prediction and Design of Uropathogenic *Escherichia coli* Alpha-Hemolysin Generate a Soluble and Hemolytic Recombinant Toxin

**DOI:** 10.3390/microorganisms10010172

**Published:** 2022-01-14

**Authors:** Bruna De Lucca Caetano, Marta de Oliveira Domingos, Miriam Aparecida da Silva, Jessika Cristina Alves da Silva, Juliana Moutinho Polatto, Fabio Montoni, Leo Kei Iwai, Daniel Carvalho Pimenta, Hugo Vigerelli, Paulo Cesar Gomes Vieira, Rita de Cassia Ruiz, José Salvatore Patané, Roxane Maria Fontes Piazza

**Affiliations:** 1 Laboratório de Bacteriologia, Instituto Butantan, Av. Vital Brazil, São Paulo 1500-05503-900, SP, Brazil; bruna3caetano@gmail.com (B.D.L.C.); marta.domingos@butantan.gov.br (M.d.O.D.); miriam.silva@butantan.gov.br (M.A.d.S.); jessika.cristina4@hotmail.com (J.C.A.d.S.); juliana.yassuda@butantan.gov.br (J.M.P.); paulo.vieira@butantan.gov.br (P.C.G.V.); rita.ruiz@butantan.gov.br (R.d.C.R.); 2 Laboratório de Toxinologia Aplicada, Instituto Butantan, Av. Vital Brazil, São Paulo 1500-05503-900, SP, Brazil; fabio.montoni@hotmail.com (F.M.); leo.iwai@butantan.gov.br (L.K.I.); 3 Laboratório de Biofísica e Bioquímica, Instituto Butantan, Av. Vital Brazil, São Paulo 1500-05503-900, SP, Brazil; dcpimenta@butantan.gov.br (D.C.P.); hugovigerelli@gmail.com (H.V.); 4 Laboratório de Ciclo Celular, Instituto Butantan, Av. Vital Brazil, São Paulo 1500-05503-900, SP, Brazil

**Keywords:** bacterial toxins, uropathogenic *Escherichia coli*, alpha-hemolysin, in silico prediction

## Abstract

The secretion of α-hemolysin by uropathogenic *Escherichia coli* (UPEC) is commonly associated with the severity of urinary tract infections, which makes it a predictor of poor prognosis among patients. Accordingly, this toxin has become a target for diagnostic tests and therapeutic interventions. However, there are several obstacles associated with the process of α-hemolysin purification, therefore limiting its utilization in scientific investigations. In order to overcome the problems associated with α-hemolysin expression, after in silico prediction, a 20.48 kDa soluble α-hemolysin recombinant denoted rHlyA was constructed. This recombinant is composed by a 182 amino acid sequence localized in the aa542–723 region of the toxin molecule. The antigenic determinants of the rHlyA were estimated by bioinformatics analysis taking into consideration the tertiary form of the toxin, epitope analysis tools, and solubility inference. The results indicated that rHlyA has three antigenic domains localized in the aa555–565, aa600–610, and aa674–717 regions. Functional investigation of rHlyA demonstrated that it has hemolytic activity against sheep red cells, but no cytotoxic effect against epithelial bladder cells. In summary, the results obtained in this study indicate that rHlyA is a soluble recombinant protein that can be used as a tool in studies that aim to understand the mechanisms involved in the hemolytic and cytotoxic activities of α-hemolysin produced by UPEC. In addition, rHlyA can be applied to generate monoclonal and/or polyclonal antibodies that can be utilized in the development of diagnostic tests and therapeutic interventions.

## 1. Introduction

Urinary tract infection (UTI) induced by uropathogenic *Escherichia coli* (UPEC) is a condition that affects more than half of women around the world and causes significant economic and social burden. The majority of UTIs are characterized by acute uncomplicated cystitis. However, in some cases, the infection can ascend to the kidneys and induce pyelonephritis. It has been demonstrated that 78% of the cases of pyelonephritis induced by UPEC were associated with the presence of α-hemolysin (HlyA), indicating that the production/secretion of HlyA is correlated with the severity of the disease [1].

HlyA is a pore-forming toxin that belongs to the repeats-in-toxin (RTX) family whose activities depends on calcium [1,2]. The interaction of HlyA with the target cell membrane has broad specificity and is devoid of a specific protein receptor [2]. The necessity of oligomerization to pore formation is also a matter of controversy since HlyA has been recovered from targeted membranes only as monomers [2].

Several studies have also demonstrated that HlyA can induce pore formation in different types of cells such as lymphocytes, red blood cells, and epithelial cells, and thus disrupt the cell osmotic balance [1]. 

It has been acknowledged that HlyA participates in a series of cell signaling pathways during UTI. For instance, it has been demonstrated that in UPEC infection, HlyA is essential for the induction of human macrophage cell death and IL-1β release by NLRP3 activation [3]. It has also been shown that HlyA can inhibit the activation of protein kinase B (Akt), a molecule involved in the regulation of cellular metabolism, cell proliferation, and inflammatory responses [4].

Despite the importance of the HlyA in cell activation during UPEC infection, studies about HlyA are scarce because of the difficulties involved in the process of its purification [5]. The methods that generate better toxin yield generate HlyA in insoluble fractions and in inclusion bodies that require additional steps to be obtained [6,7].

Recently, several studies have been used in silico platforms in order to predict the behavior of toxins in vivo [8]. There are also available several computational programs able to determine the tridimensional and the quaternary form of protein complexes (e.g., https://services.healthtech.dtu.dk/, accessed on 14 February 2021). In addition, the in silico approaches for the prediction and analysis of proteins can estimate the solubility of a recombinant and evaluate whether it would be recognized by antibodies and/or cells of the immune system [9]. These procedures are extremely important in several areas including the development of vaccines, therapies, and diagnostics [10]. In this study, using in silico technology, a soluble intermediate form of HlyA was constructed, designed to present antigenic determinant epitopes of the tertiary structure of the toxin. The results indicate that the recombinant created in this study (rHlyA) can be easily obtained and used as a tool to develop UPEC diagnostic tests able to predict the prognosis of the infection.

## 2. Materials and Methods

### 2.1. Bacterial Strains and Growth Conditions

The bacterial strains used in this study were the hemolytic uropathogenic *E. coli* O2:H1 (DV78) and the non-hemolytic *E. coli* DH5α [11]. These *E. coli* strains belong to the bacterial collection of the Bacteriology Laboratory of Butantan Institute. Prior to hemolysis test, 30 μL of bacterial culture was added in triplicate to 3 mL of trypticase soy broth (TSB) and incubated overnight at 37 °C for 18 h.

### 2.2. Cell Culture

Epithelial cells from human bladder (5637 cells—ATCC^®^ HTB9™) were grown in RPMI media (Cultilab, Campinas, São Paulo, Brazil) supplemented with 10% fetal bovine serum (FBS) (Cultilab, Campinas, São Paulo, Brazil). These cells were grown in a humidified atmosphere with 5% CO_2_, at 37 °C. The subcultures were harvested with trypsin-EDTA (Gibco) pH 7.4, washed, and distributed at 5 × 10^4^ cells/well in 96-well culture plates (Corning Incorporated Costar^®^—New York, NY, USA) 24 h before carrying out the cell viability assays.

### 2.3. Design and Construction of Immunogenic Recombinant HlyA

The rationale of this methodology was based on the knowledge of cloning techniques and heterologous protein expression, where it is known that smaller sequences, representing proteins of approximately 20 kDa, are more easily obtained. For the in silico analysis, the software Phyre^2^ (http://www.sbg.bio.ic.ac.uk/~phyre2/, accessed on 14 February 2021), SwissModel (https://swissmodel.expasy.org/, accessed on 14 February 2021) and I-TASSER (https://zhanglab.ccmb.med.umich.edu/I-TASSER/, accessed on 14 February 2021) were used to unveil the tertiary structure of proteins, since there is no such annotation in the literature. Subsequently, the 3Drefiner program (http://sysbio.rnet.missouri.edu/3Drefine/, accessed on 14 February 2021) was used to refine the three-dimensional structures obtained. 

General data on partial proteins were obtained by the ProtParam program (https://web.expasy.org/protparam/, accessed on 14 February 2021). Briefly, the signal peptide was first identified and removed using SignalP v5.0 [12]. Subsequently the HlyA sequence was subjected to estimation of tertiary structure in Phyre2 [13] SwissModel [14] and I-TASSER [15]. The scores for all models obtained were assessed by C-score in I-TASSER, and the largest scoring model was chosen as the best candidate for tertiary structure. Further refinement of this structure was performed in 3Drefiner [16]. Antigenicity estimation was performed in BepiPred v2.0 [17]. The most soluble and largest epitopes were selected for laboratory testing using ProteinSol (https://protein-sol.manchester.ac.uk/, accessed on 14 February 2021), ProtScale (https://web.expasy.org/protscale/, accessed on 14 February 2021) and SOLart (https://mybiosoftware.com/solart-1-0-protein-solubility-prediction.html, accessed on 14 February 2021).

The coding gene sequence for these regions of *hlyA* was sent for synthesis (at FastBio Ltd.a., Supera Parque, Brazil) in a pET30a(+) expression vector containing the restriction enzymes NdeI and NotI, for the possibility of extracting and altering the plasmid used. Therefore, providing the construction pET30-hlyA-His generating a recombinant hemolysin, herein nominated rHlyA.

### 2.4. Expression and Purification of the Recombinant Protein

Chemically competent *E. coli* BL21 (DE3) [18] was transfected. Initially, 1 μL of plasmid was incubated with 2 μL of 5× KCM buffer (0.5 M KCl, 0.15 M CaCl_2_, and 0.25 M MgCl_2_) and 7 μL of deionized water on ice for 5 min, followed by the addition of 10 μL of chemically competent cells; after 20 min, the solution was transferred to 24 °C for 10 min. Subsequently, 200 μL of Lysogeny broth (LB) culture medium were added and the sample was incubated at 37 °C for 1 h. The cells were then streaked on an LB agar plate containing 100 μg/mL of ampicillin and/or 12 μg/mL of tetracycline and incubated at 37 °C for 18 h.

Bacteria were grown in 200 mL of LB medium supplemented with the same antibiotic at 37 °C for 4 h and stirring at 250 rpm. When in log phase, measured by optical density (OD) at 600 nm, with values between 0.6 and 0.8 we added isopropyl-β-D-galactoside (IPTG) at 0.1 mM final concentration to induce protein expression. After incubation at 30 °C for 18 h at 250 rpm, cells were separated in a 5804 R centrifuge (Eppendorf, Hamburg, Germany) at 10,000× *g* for 10 min and the supernatant was discarded. The pellet was resuspended in 20 mL of lysis buffer (182 mL modified PBS buffer, 0.05 M NaH_2_PO_4_, 0.3 M NaCl pH 8.0; 40 mg lysozyme; 1% Triton X-100; 1% MgCl_2_ 2 M; 1% PIC, 0.1 M PMSF, 0.1 M benzamidine; 1 μL benzonase) under gentle stirring at 4 °C for 30 min, followed by disruption of cell suspension for 3 cycles of 10 min at amplitude 30 and cycle 9 (Bandelin Sonopuls, Berlin, Germany). The lysate was centrifuged at 10,000× *g* for 30 min.

Protein expression was assessed by 12% polyacrylamide gel electrophoresis (SDS-PAGE), where 15 μL of the 2-mercaptoethanol-treated protein samples were heat-denatured at 100 °C for 5 min and applied on a 12% SDS-PAGE gel. 

A column containing the 5 mL volume of Nickel-loaded Sepharose (Ni^2+^) was used with the ÄKTAprime plus chromatography system (GE Healthcare, Chicago, IL, USA). Purification was performed by applying the supernatant after lysis by sonication to the previously equilibrated column with binding buffer (20 mM Tris-HCl, pH 7.4, containing 5 mM imidazole and 0.5 M NaCl). Elution was performed with an elution buffer (20 mM Tris-HCl, pH 7.4, containing 1 M imidazole and 0.5 M NaCl). After elution, the protein was dialyzed against phosphate-buffered saline (PBS) (0.01 M, pH 7.2) with three buffer changes of 3 h/each. Subsequently, the proteins were concentrated in the presence of polyethylene glycol 6000 (PEG 6000). To confirm purification, 12% polyacrylamide gel electrophoresis (SDS-PAGE) was performed.

### 2.5. Mass Spectrometry Analysis

Liquid chromatography–mass spectrometry (LC-MS) analyses were performed using an Electrospray-Ion Trap Time of Flight system (ESI-IT-TOF) (Shimadzu Co., Kyoto, Japan) equipped with a binary Ultra-Fast Liquid Chromatography system (UFLC) (20 A Prominence, Shimadzu) at the Laboratory of Biochemistry and Biophysics of the Butantan Institute (São Paulo, Brazil). First, each band sample was lyophilized, resuspended in 50 µL of 0.1% acetic acid, and loaded on a C18 column (Discovery C18, 5 µm; 50 × 2.1 mm) in a binary solvent system: (A2) water/acetic acid (999/1, *v*/*v*) and (B2) ACN/water/acetic acid (900/99/1, *v*/*v*/*v*). The column was eluted at a constant flow rate of 0.2 mL. min−1 with a 5 to 70% gradient of solvent B2 over 35 min. The eluates were monitored by a Shimadzu SPD-M20A PDA detector before introduction into the mass spectrometer. The interface voltage was adjusted to 4.5 kV, and the capillary voltage was 1.76 kV at 200 °C. MS spectra were acquired in positive mode and collected in the 80–2000 mass charge (m/z) range. MS/MS spectra were collected in the 50–1950 m/z range. Instrument control, data acquisition, and data processing were performed with LabSolutions (LCMSsolution 3.60.361 version, Shimadzu). Proteomic analysis was performed using the Mascot Server (ion search) in-house version (2.4) and Peaks Studio V7 (Bioinformatics Solutions, Inc., Waterloo, Canada). The following parameters were adjusted for the search: parent mass and fragment mass error tolerance: 0.1 Da; enzyme: trypsin; fixed modification: carbamidomethylation; variable modification: methionine oxidation; precursor mass search type: monoisotopic; max missed cleavages: 3; non-specific cleavages: 1; database: SwissProt.

### 2.6. Hemolysis Assay

Blood-agar culture plates were prepared according to Beutin et al. [19]. Briefly, 1.5 g of (Tryptic Soy Agar (TSA) plus 10 mM solution of CaCl_2_ or in the absence of CaCl_2_ were autoclaved. When the temperature of the agar dropped to 45 °C, sheep red cells previously washed three times in PBS pH 7.2 were then added to the agar until a final concentration of 5%. The agar was dispensed to Petri dish plates (20 mL per plate), left to solidify, and kept at 4 °C until use. Then, 4 µL of each bacterial culture was added to the blood agar plates. In parallel, 40 µL of purified rHlyA was also added to the blood agar plates. The plates were then incubated at 37 °C for 18 h and the presence of hemolysis was determined by the formation of a halo of lysed erythrocytes around either of the bacterial growth or in the point where rHlyA was dotted.

### 2.7. Cell Assay

Different concentrations of rHlyA (25, 50, or 100 µg/mL) were incubated overnight to 5637 cells and then subjected to the following methods. MTT assay, based on the metabolic reduction of 3-(4,5-dimethylthiazol-2-yl)-5-(3-carboxymethoxyphenyl) -2-(4-sulfophenyl) -2H-tetrazolium by mitochondrial enzyme activity of viable cells was determined according to the manufacturer’s instructions (Sigma-Aldrich, St. Louis, MO, USA). Crystal Violet that binds to protein and DNA detects cells that have remained attached, post-treatment. After incubation with rHlyA the plate was washed 2× with PBS and fixed with methanol for 10 min. Afterwards, the cells were again washed 2× with PBS, stained with Crystal violet solution, for 10 min. After 2 new washes, cultures were destained with 100 µL of 0.1 M sodium citrate for 30 min. The positive control for cell cytotoxicity consisted in treatment with 0.1% triton-X100 for 30 min. Absorbance was determined at 550 nm in an ELISA reader Multiskan^®^EX (Thermo Fisher Scientific, Waltham, MA, USA) [20]. The results are representative of experiments performed in triplicate. Statistical analysis was performed using GraphPad Prism 6.0 (San Diego, CA, USA), One Way ANOVA. Results were considered statistically significant when *p* ≤ 0.05.

## 3. Results

### 3.1. In Silico Design and Prediction

This work was based on an in silico analysis that aimed to identify immunogenic regions of hemolysin and further select small DNA sequences that could be commercially obtained as synthetic genes for cloning and expression. The selection of these regions was performed based on the results of the linear prediction of antigenicity, which provides the likely amino acid sequence forming an epitope on the protein. With these data, it was possible to choose a region containing the most probable epitopes for the production of new synthetic genes. Moreover, the solubility of each portion was evaluated, as it will be a protein expressed in a heterologous system. Different software was used to ensure the probability that the region was soluble, and in all of them, the result was similar, being an indication that the protein can be expressed in an optimized way.

**Figure 1:**
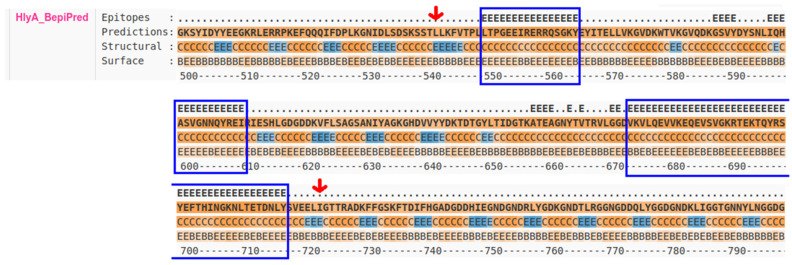
Prediction of epitopes by the BepiPred program. The predicted epitopes in HlyA are highlighted with blue rectangles. The arrows indicate the start and the end of the selected amino acid sequence for cloning and expression. E: an amino acid likely in an inferred epitope segment; H: α-helix; E: β-sheet; C: coil; B (and E in last line): buried/exposed.

Phyre-2 and SwissModel estimated similar models, whilst I-TASSER could not identify a structure including more than a few amino acids. Regarding the models inferred by the two former software’s, the Phyre^2^ inference had the highest C-score, and this structure was used for subsequent analysis. To obtain the linear epitopes, we used BepiPred on the primary structure of the HlyA. Six epitopes were selected a priori, based on the solubility of each epitope. Out of those, we narrowed it down to three epitopes, regarding their spatial proximity, aiming to generate a highly immunogenic recombinant protein (Figure 1, Table 1).

**Figure 2:**
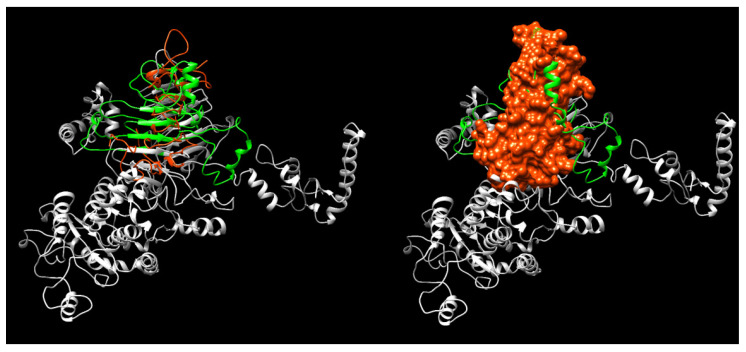
Prediction of α-hemolysin quaternary structure. rHlyA structure aligned to the complete HlyA protein. (Left) Ribbon structures (**green:** actual region on the inferred whole HlyA; **red:** inferred rHlyA structure); (Right) rHlyA molecule surface.

This prediction allowed us to reach a quaternary structure, in which the parts in red indicate the predicted epitopes (Figure 2). 

Accordingly, it was selected a 182 amino acid sequence in the (aa542–aa723) region of the HlyA toxin for the construction of a soluble antigenic 20.48 kDa recombinant protein (Table 2).

### 3.2. Cloning, Expression, and Purification

**Figure 3:**
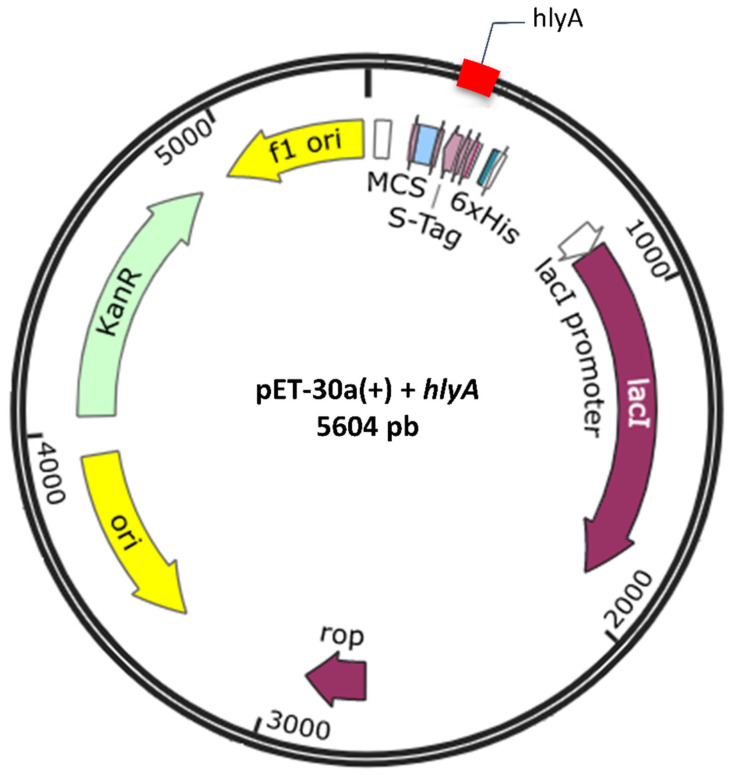
Constructed DNA plasmid encoding *hlyA* gene.

The coding gene sequence for these regions was sent for synthesis (at FastBio Ltd.a.) in a pET30a(+) expression vector (Figure 3). 

**Figure 4:**
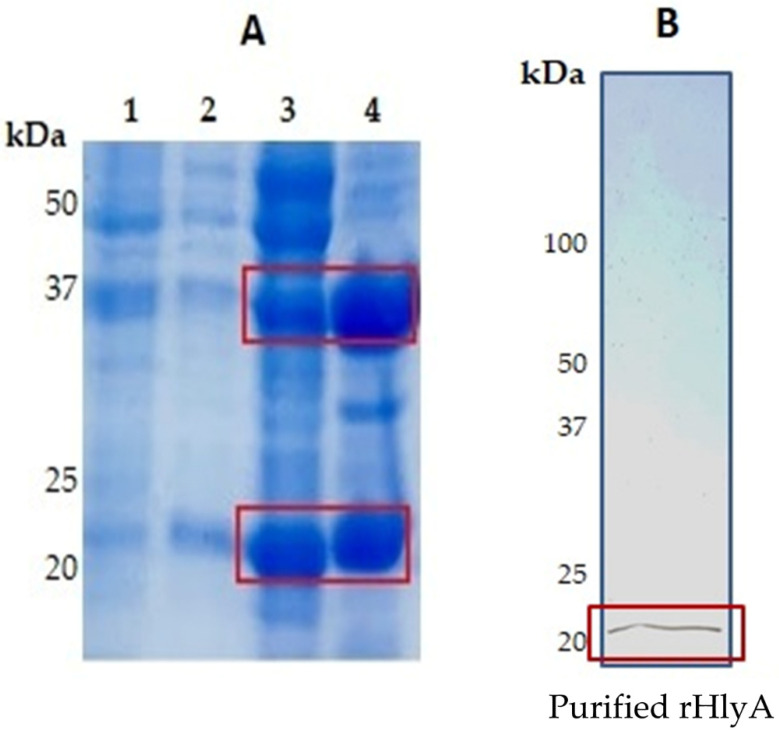
Twelve percent SDS-PAGE of the HlyA antigenic recombinant constructs: (**A**). SDS/PAGE Coomassie blue-stained: 1. Pre-induced; 2. Post-induced; 3. Disrupted supernatant; 4. Disrupted sediment. (**B**). SDS/PAGE silver-stained: purified rHlyA from affinity chromatography purification [(Nickel-loaded Sepharose (Ni^2+^)].

After insertion of the synthetic gene in *E. coli* bacteria C43(DE3), BL21 Star (DE3), and BL21(DE3) pLysS and expression, the proteins were analyzed by SDS-PAGE to assess whether they were present after bacterial growth. Protein expression occurred only in BL21(DE3) pLysS, resulting in the presence of two bands, one with molecular mass of approximately 20.48 kDa and the other with 37 kDa (Figure 4A). After mass spectrometry the proteomic analysis of the bands using UNIPROTB database indicates that the protein with molecular mass of 20.48 kDa is associated with *E. coli* alpha-hemolysin (UniprotkB: P09983, HLYAC-ECOLX), whereas the 37-kDa protein is a glyceraldehyde-3-phosphate dehydrogenase (UniprotKB: P0A1P0, G3p_SALTY) (Table 3). It was also observed that both proteins are present in the supernatant and sediment of transformed BL21(DE3) pLysS lysate. The 37-kDa proteins, however, were more expressed in the sediment (Figure 4A). The purified recombinant HlyA is shown after purification by chromatography in Nickel-loaded Sepharose (Figure 4B).

### 3.3. Proteomic Processing

**Figure 5:**
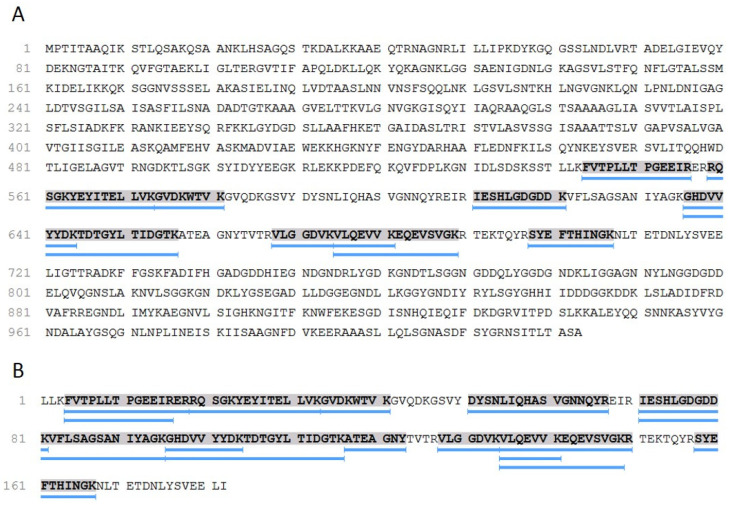
(**A**) Coverage map representing the proteomic identification of Hemolysin (P09983), and (**B**) recombinant HlyA, according to Peaks Studio analyses. Underlines in blue represent the proteomically-matched peptides over the deposited sequence from *Escherichia coli*.

The 12% SDS-PAGE gel band corresponding to the HlyA protein-like molecular mass component was proteomically prepared and analyzed by mass spectrometry. Four peptides (Table 3) matched to hemolysin (P09983), with 10% coverage (Figure 5A), considering the original protein sequence (~110 kDa). However, limiting the analyses to the recombinant sequence (HlyA), the coverage rises to 59% (Figure 5A). Underlined in blue, represent the proteolytically combined peptides on the deposited sequence of *Escherichia coli.* Supplemental Figure S1 presents the alignment between P09983 and alpha-hemolysin, indicating that the peptides matched to P09983 are comprised between positions 543 and 717 of the whole hemolysin.

### 3.4. Phenotypical Features of HlyA

#### 3.4.1. Hemolytic Activity

**Figure 6:**
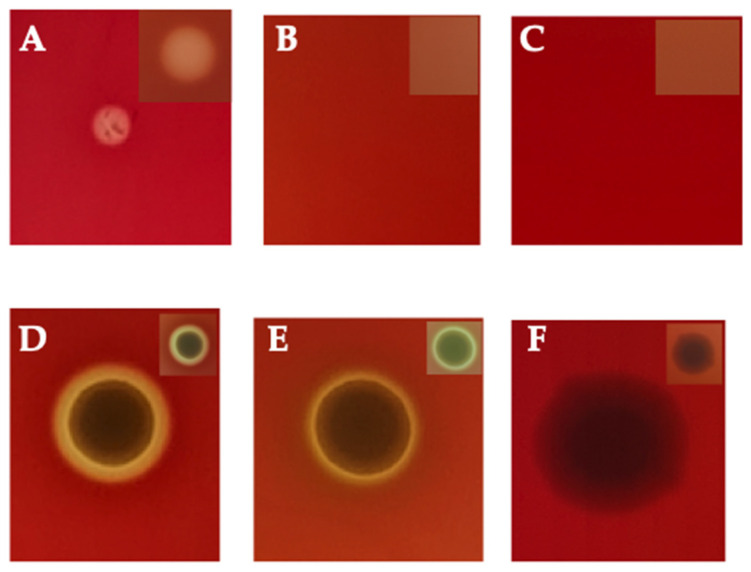
Hemolytic profile in blood agar hemolysis assay in the presence and absence of CaCl_2_: (**A**). rHlyA in the presence of CaCl_2_; (**B**). rHlyA in the absence of CaCl_2_; (**C**). PBS 0.01 M pH 7.2; (**D**). Hemolysin-producing UPEC (DV73) strain in the presence of CaCl_2_; (**E**). Hemolysin-producing UPEC (DV73) strain in the absence of CaCl_2_; (**F**). Non-hemolysin-producing UPEC strain (DH5α). The figures represent the amplified pictures inserted in the top right corner.

The functionality of rHlyA was first checked by hemolytic activity in blood agar, either in the presence or in absence of CaCl_2_. The results showed clearly that rHlyA causes hemolysis in blood agar in the presence of CaCl_2,_ but not in its absence, which was expected since its activity is calcium-dependent (Figure 6A,B). This activity was due solely to the purified toxin, since the buffer in which it was contained, no effect was observed (Figure 6C). UPEC DV73 also induced hemolysis in the presence of CaCl_2_. However, its hemolytic activity diminished in the absence of CaCl_2_, but did not disappear, since, besides HlyA, this UPEC strain can produce other toxins with hemolytic activity (Figure 6D,E). DH5α was employed as a non-producing one (Figure 6F). 

#### 3.4.2. Cytotoxic Activity

**Figure 7:**
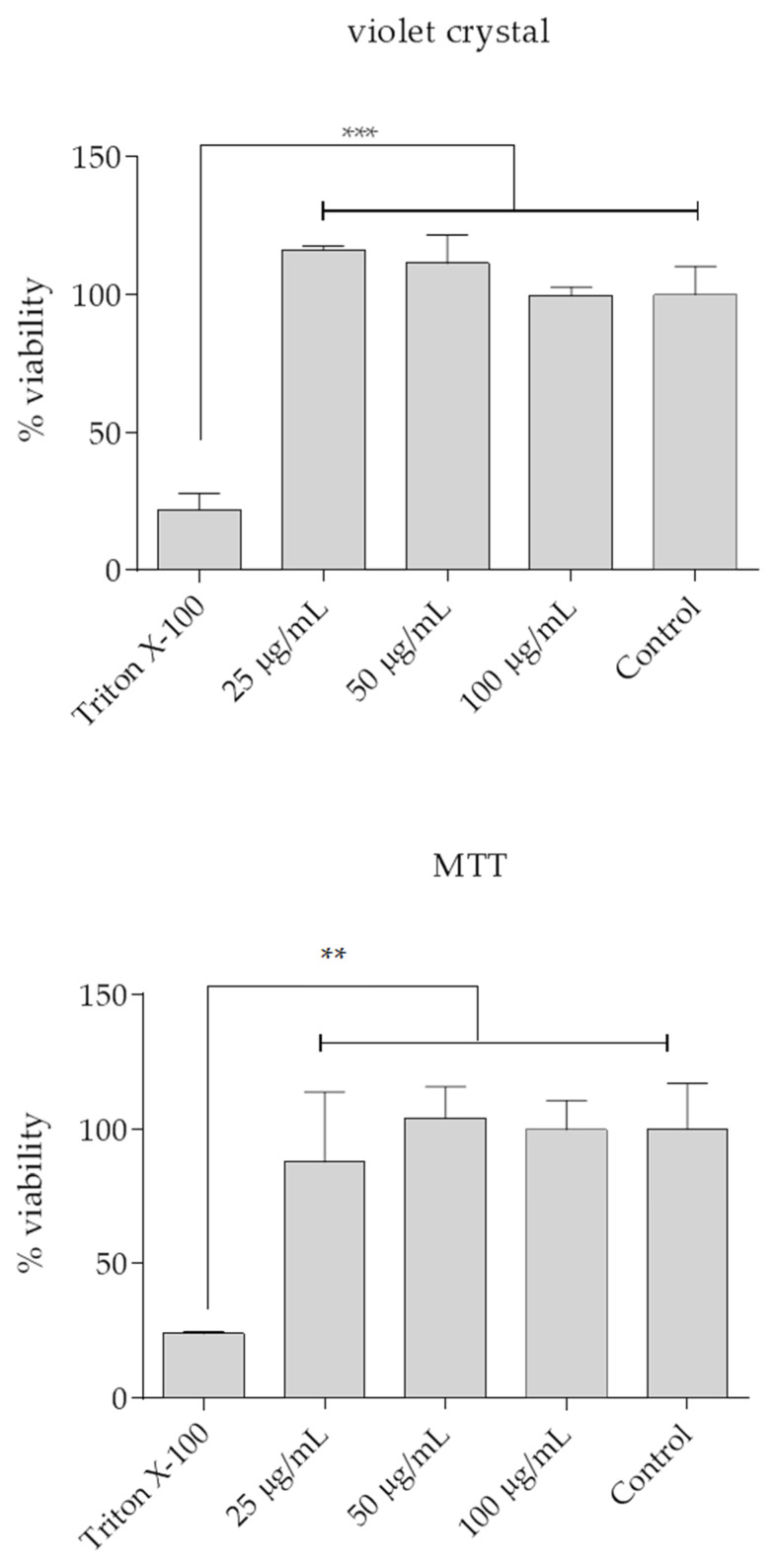
The cultures were subjected to analysis by Violet Crystal and MTT assays. In both assays, no significant differences were observed between the viability of cells treated with the three concentrations of rHlyA and the positive control *p* > 0.05. Cell viability was significantly reduced only in the negative control (Triton X-100) when compared to the different groups. *** *p* ≤ 0.001 and ** *p* ≤ 0.01, Crystal Violet and MTT, respectively.

To assess whether rHlyA had any cytotoxic effect, human cells of 5637 lineage, derived from the urinary bladder, were incubated with 25, 50, and 100 µg/mL of rHlyA for 18 h. Cell viability was investigated under two parameters, cell detachment by Crystal Violet assay and mitochondrial metabolism by MTT. The results of the Crystal Violet and MTT assays are shown in Figure 7. The effects of rHlyA on cell viability in both methods, at the three concentrations tested, were statistically similar and comparable to the negative control, i.e., cells without any treatment (*p* ≥ 0.05). Triton X-100 was employed as positive control for the cytotoxic effect compared to the different groups (*p* ≤ 0.001), as expected.

## 4. Discussion

The alpha-hemolysin produced by UPEC is secreted to the extracellular milieu by a type I secretion pathway consisting of HlyB, HlyD, and TolC proteins [21]. The toxin can be either detected free in culture supernatants or associated with bacterial outer membranes and outer membrane vesicles [22,23]. Alpha-hemolysin can also use the CD14/LPS-binding protein complex as a delivery vehicle to mammalian cell membranes [24].

In its secreted form, alpha-hemolysin has cytolytic and/or cytotoxic activity against a wide variety of cells. However, controversial results have been published about the mechanism involved in HlyA pore formation. For instance, some studies have demonstrated that HlyA can form pores in monomeric form, whereas others imply the importance of molecular oligomerization [1]. All the data obtained in years of research remain elusive since no imaging technology has ever been able to capture alpha-hemolysin during pore formation [1].

The difficulties related to the studies of HlyA activities are increased by the methodology used to purify the whole toxin, since it is a difficult and time-consuming process that generates a low yield of purified protein [6,7]. To overcome this problem, after employing bioinformatics tools, a recombinant hemolysin named rHlyA was constructed here by selecting amino acid regions that could ensure the solubility of the molecule. Therefore, a 182 amino acid sequence localized in the aa542–723 region of the protein was selected. The selected sequence generated a soluble recombinant protein with molecular mass of 20.48 kDa. Bioinformatics’ tools aimed at inferring protein folding (in the present case, HlyA) are important due to the difficulties mentioned above; knowing the folding of a relevant segment of the whole HlyA molecule can be fundamental to validate previous biochemical characteristics, and even to infer possible new ones, such as interactions/behavior associated with expression, transcription, translation, and functional specificities (such as regions influencing the likelihood of infection) otherwise hard to obtain in a wet lab [25]. Furthermore, immunoinformatics approaches are extremely useful at selecting viable candidates for both diagnostic (as shown here regarding linear epitope estimation in HlyA), and even its putative use as peptide-based treatments aimed at controlling specific infections [26].

Since the hemolytic and/or cytotoxic domains of the HlyA toxin have not been clearly described, rHlyA was also designed to contain domains with proteolytic activities. The results demonstrated that rHlyA is hemolytic, suggesting that the hemolytic domain of the whole toxin is located in the aa542–723 region of the protein. A similar result was achieved by Ristow and Welch [1] using a chimera of another RTX toxin, the leucotoxinA (LktA), whose hemolytic domain was found between the aa563–739 region [1].

Despite its hemolytic activity, rHlyA presented no cytotoxic effect against human bladder cells, suggesting that the aa542–723 region of the toxin has hemolytic but no cytotoxic activity. These results are supported by the work of Forestier and Welch [27], who demonstrated that a chimera of HlyA containing the aa1–739 region presented hemolytic but not cytotoxic activity. In addition, the use of the monoclonal antibody D12 that binds to the region aa673–726 is able to block the hemolytic activity of the toxin [28]. It is worth noting that the antigenic sequence aa674–717 of rHlyA is equivalent to the domain recognized by the monoclonal antibody D12 [28]. This result indicates that rHlyA should be able to generate monoclonal and/or polyclonal antibodies able to inhibit the hemolytic activity of the α-hemolysin.

Considering that the two other antigenic determinants of rHlyA are localized in an amino acid region described as responsible for the hemolytic activity of the toxin, the generation of antibodies against these epitopes may also have the ability to inhibit the hemolytic effect of α-hemolysin [1].

In summary, the data obtained in this study suggested that rHlyA can generate monoclonal and/or polyclonal antibodies that can be used as tools in experiments to understand the mechanisms that underlie the functional activities of the α-hemolysin produced by UPEC, as well as diagnostic tools to predict the prognosis of UPEC infections. Accordingly, experiments are being conducted to confirm the ability of rHlyA to generate neutralizing antibodies against alpha-hemolysin.

## Figures and Tables

**Table 1:** Epitope prediction screening from the region (542 aa-723 aa) of alpha-hemolysin.

Peptide Sequence		Length	Start	End	pI
1	LTPGEEIRERRQSGKY	16	550	565	8.59
2	ASVGNNQYREI	11	600	610	6.05
3	VKVLQEVVKEQEVSVGKRTEKTQYRSYEFTHINGKNLTETDNLY	44	674	717	6.76

pI—isoelectric point.

**Table 2:** Alpha-hemolysin antigenic region sequence.

Protein	Sequence	Length	Molecular Weight
Alpha-hemolysin	LLKFVTPLLTPGEEIRERRQSGKYEYITELLVKGVDKWTVKGVQDKGSVYDYSNLIQHASVGNNQYREIRIESHLGDGDDKVFLSAGSANIYAGKGHDVVYYDKTDTGYLTIDGTKATEAGNYTVTRVLGGDVKVLQEVVKEQEVSVGKRTEKTQYRSYEFTHINGKNLTETDNLYSVEELI	182	20.48 kDa

**Table 3:** Proteins identified by mass spectrometry.

Description	Accession	−10lgP	#Peptides	Coverage (%)	Avg. Mass	Organism
Hemolysin, chromosomal	P09983	111.40	10	10	109,867	*Escherichia coli*
Chloramphenicol acetyltransferase	P62577	88.31	7	17	25,663	*Escherichia coli*
50S ribosomal protein	B61233	28.38	1	3	22,087	*Escherichia coli*
Hly A (recombinant)	-	222.24	11	59	20,480	*Escherichia coli*

## Data Availability

The data presented in this study are available on request from the corresponding authors. The data are not available in the repository of the Butantan Institute (https://repositorio.butantan.gov.br (accessed on 13 December 2021)).

## Supplementary Materials

The following are available online at https://www.mdpi.com/article/10.3390/microorganisms10010172/s1, Figure S1: Alignment between P09983 and alpha-hemolysin, indicating that the peptides matched to P09983 are between positions 543 and 717 of the whole hemolysin.
