# Supplementary material for: In Silico Prediction and Design of Uropathogenic Escherichia coli Alpha-Hemolysin Generate a Soluble and Hemolytic Recombinant Toxin
Source: Microorganisms. 2022 Jan 14;10(1):172. doi: 10.3390/microorganisms10010172 (PMC8778037; doi:10.3390/microorganisms10010172)
Supplement: Supplementary file 1 [file microorganisms-10-00172-s001.zip › microorganisms-1530493-supplementary.pdf]

```
CLUSTAL O(1.2.4) multiple sequence alignment


HlyA            ------------------------------------------------------------ 0
sp|P09983|      MPTITAAQIKSTLQSAKQSAANKLHSAGQSTKDALKKAAEQTRNAGNRLILLIPKDYKGQ 60


HlyA            ------------------------------------------------------------ 0
sp|P09983|      GSSLNDLVRTADELGIEVQYDEKNGTAITKQVFGTAEKLIGLTERGVTIFAPQLDKLLQK 120


HlyA            ------------------------------------------------------------ 0
sp|P09983|      YQKAGNKLGGSAENIGDNLGKAGSVLSTFQNFLGTALSSMKIDELIKKQKSGGNVSSSEL 180


HlyA            ------------------------------------------------------------ 0
sp|P09983|      AKASIELINQLVDTAASLNNVNSFSQQLNKLGSVLSNTKHLNGVGNKLQNLPNLDNIGAG 240


HlyA            ------------------------------------------------------------ 0
sp|P09983|      LDTVSGILSAISASFILSNADADTGTKAAAGVELTTKVLGNVGKGISQYIIAQRAAQGLS 300


HlyA            ------------------------------------------------------------ 0
sp|P09983|      TSAAAAGLIASVVTLAISPLSFLSIADKFKRANKIEEYSQRFKKLGYDGDSLLAAFHKET 360


HlyA            ------------------------------------------------------------ 0
sp|P09983|      GAIDASLTRISTVLASVSSGISAAATTSLVGAPVSALVGAVTGIISGILEASKQAMFEHV 420


HlyA            ------------------------------------------------------------ 0
sp|P09983|      ASKMADVIAEWEKKHGKNYFENGYDARHAAFLEDNFKILSQYNKEYSVERSVLITQQHWD 480


HlyA            ------------------------------------------------------------ 0
sp|P09983|      TLIGELAGVTRNGDKTLSGKSYIDYYEEGKRLEKKPDEFQKQVFDPLKGNIDLSDSKSST 540


HlyA            LLKFVTPLLTPGEEIRERRQSGKYEYITELLVKGVDKWTVKGVQDKGSVYDYSNLIQHAS 60
sp|P09983|      LLKFVTPLLTPGEEIRERRQSGKYEYITELLVKGVDKWTVKGVQDKGSVYDYSNLIQHAS 600
                ************************************************************

HlyA            VGNNQYREIRIESHLGDGDDKVFLSAGSANIYAGKGHDVVYYDKTDTGYLTIDGTKATEA 120
sp|P09983|      VGNNQYREIRIESHLGDGDDKVFLSAGSANIYAGKGHDVVYYDKTDTGYLTIDGTKATEA 660
                ************************************************************

HlyA            GNYTVTRVLGGDVKVLQEVVKEQEVSVGKRTEKTQYRSYEFTHINGKNLTETDNLYSVEE 180
sp|P09983|      GNYTVTRVLGGDVKVLQEVVKEQEVSVGKRTEKTQYRSYEFTHINGKNLTETDNLYSVEE 720
                ************************************************************

HlyA            LI---------------------------------------------------------- 182
sp|P09983|      LIGTTRADKFFGSKFADIFHGADGDDHIEGNDGNDRLYGDKGNDTLSGGNGDDQLYGGDG 780
                **

HlyA            ------------------------------------------------------------ 182
sp|P09983|      NDKLIGGAGNNYLNGGDGDDELQVQGNSLAKNVLSGGKGNDKLYGSEGADLLDGGEGNDL 840


HlyA            ------------------------------------------------------------ 182
sp|P09983|      LKGGYGNDIYRYLSGYGHHIIDDDGGKDDKLSLADIDFRDVAFRREGNDLIMYKAEGNVL 900


HlyA            ------------------------------------------------------------ 182
sp|P09983|      SIGHKNGITFKNWFEKESGDISNHQIEQIFDKDGRVITPDSLKKALEYQQSNNKASYVYG 960


HlyA            ------------------------------------------------------------ 182
sp|P09983|      NDALAYGSQGNLNPLINEISKIISAAGNFDVKEERAAASLLQLSGNASDFSYGRNSITLT 1020


HlyA            --- 182
sp|P09983|      ASA 1023
```

Figure S1. Alignment between P09983 and alpha-hemolysin, indicating that the peptides matched to P09983 are between positions 543 and 717 of the whole hemolysin.
